# Naive poison frog tadpoles use bi-modal cues to avoid insect predators but not heterospecific predatory tadpoles

**DOI:** 10.1242/jeb.243647

**Published:** 2021-12-16

**Authors:** Birgit Szabo, Rosanna Mangione, Matthias Rath, Andrius Pašukonis, Stephan A. Reber, Jinook Oh, Max Ringler, Eva Ringler

**Affiliations:** 1 Division of Behavioural Ecology, Institute of Ecology and Evolution, University of Bern, Wohlenstrasse 50a, 3032 Hinterkappelen, Switzerland; 2 Department of Behavioral and Cognitive Biology, University of Vienna, 1030 Vienna, Austria; 3 CEFE, Université de Montpellier, CNRS, EPHE, IRD, 34293 Montpellier 5, France; 4 Lund University Cognitive Science, Lund University, 223 62 Lund, Sweden; 5 Messerli Research Institute, University of Veterinary Medicine Vienna, 1210 Vienna, Austria; 6 Cremer Group, Institute of Science and Technology Austria, 3400 Klosterneuburg, Austria; 7 Department of Evolutionary Biology, University of Vienna, 1030 Vienna, Austria; 8 Institute of Electronic Music and Acoustics, University of Music and Performing Arts Graz, 8010 Graz, Austria

**Keywords:** Anti-predator behavior, Anuran, Cross-modal integration, Innate predator detection, Cue recognition

## Abstract

For animals to survive until reproduction, it is crucial that juveniles successfully detect potential predators and respond with appropriate behavior. The recognition of cues originating from predators can be innate or learned. Cues of various modalities might be used alone or in multi-modal combinations to detect and distinguish predators but studies investigating multi-modal integration in predator avoidance are scarce. Here, we used wild, naive tadpoles of the Neotropical poison frog *Allobates femoralis* ( Boulenger, 1884) to test their reaction to cues with two modalities from two different sympatrically occurring potential predators: heterospecific predatory *Dendrobates tinctorius* tadpoles and dragonfly larvae. We presented *A. femoralis* tadpoles with olfactory or visual cues, or a combination of the two, and compared their reaction to a water control in a between-individual design. In our trials, *A. femoralis* tadpoles reacted to multi-modal stimuli (a combination of visual and chemical information) originating from dragonfly larvae with avoidance but showed no reaction to uni-modal cues or cues from heterospecific tadpoles. In addition, visual cues from conspecifics increased swimming activity while cues from predators had no effect on tadpole activity. Our results show that *A. femoralis* tadpoles can innately recognize some predators and probably need both visual and chemical information to effectively avoid them. This is the first study looking at anti-predator behavior in poison frog tadpoles. We discuss how parental care might influence the expression of predator avoidance responses in tadpoles.

## Abstract (precis)

**Summary:** Poison frog tadpoles innately recognize dragonfly larvae as dangerous using a combination of visual and olfactory cues.

## INTRODUCTION

Avoiding predation is a major goal in all organisms. Animals lacking defensive weapons or protective morphological structures have to rely on behavior to minimize detection, capture and/or consumption by predators (Petranka, 1989; Lima and Dill, 1990). Predator-induced defense behaviors are found in many species that face temporal or spatial variation in predation risk (Kats and Dill, 1998). However, life-history theory predicts that any kind of anti-predator strategy usually also incurs fitness costs (Helfman, 1989). For example, while reduced mobility and activity or hiding can considerably reduce predation risk (Lawler, 1989), it also reduces the time available for mating and foraging (Sih, 1987). Such physiological or reproductive trade-offs are found across a wide range of taxa (Tollrian and Harvell, 1999) and have probably led to the evolution of flexible anti-predator responses in animals (Reber et al., 2021).

To avoid getting captured and/or consumed, it is essential to be able to correctly identify and locate predators (Ferrari et al., 2010). Identification mechanisms can be innate (e.g. Göth, 2001; Berejikian et al., 2003; Fendt, 2006; Lau et al., 2021) or acquired and/or modulated through experience. These mechanisms might allow for a more generalized recognition of predators (Blumstein, 2006; Ferrari et al., 2007) and the matching of behavioral responses according to different predator types or to the level of perceived threat (Chivers et al., 2001). Learnt responses might, however, be most beneficial in situations in which animals face introduced predators with which they have no evolutionary history (Hettyey et al., 2016; Polo-Cavia and Gomez-Mestre, 2014). For predator recognition, animals may use cues of different modalities (e.g. visual, acoustic, chemical, electric, tactile) or a combination thereof (e.g. Amo et al., 2004, 2006; Landeira-Dabarca et al., 2019; McCormick and Manassa, 2007). Our knowledge about the contribution of different sensory systems to the recognition of particular predators to date is rather limited. In aquatic predator–prey systems, chemical signaling is considered particularly relevant, as aquatic chemical cues can usually be detected earlier and over larger distances than visual cues. Accurate localization of the source of chemical cues, however, is typically much harder than for visual cues. Thus, for an efficient anti-predator response, the combined use of both chemical and visual cues might provide optimal detection efficiency (Chivers et al., 1996; Kiesecker et al., 1999).

Amphibian larvae are an excellent model system for studying predator–prey interactions. Tadpoles are highly vulnerable to aquatic (i.e. fish, insect larvae, other tadpoles, etc.) and terrestrial (i.e. spiders, snakes, etc.) predators (Heyer et al., 1975; Gascon, 1992). Morphological (Relyea and Hoverman, 2003; Moore et al., 2004; Maher et al., 2013) and behavioral plasticity in response to variation in predation risk is known from various species (Warkentin, 1995; Relyea, 2001; van Buskirk, 2002; Laurila et al., 2004; Hammond et al., 2007; Ferrari and Chivers, 2009). The most commonly observed behavioral response to elevated predation risk in tadpoles is reduced activity (Feminella and Hawkins, 1994; van Buskirk and Arioli, 2002). Several studies have demonstrated that limited movement and/or infrequent rapid movements from one place to another can significantly increase an individual's survival probability (Lawler, 1989; Azevedo-Ramos et al., 1992). Immobility, however, presumably entails costs of reduced foraging (Azevedo-Ramos et al., 1992), resulting in a decreased energy intake that may translate into slower growth or size at metamorphosis (Laurila and Kujasalo, 1999; Relyea, 2002; Relyea and Hoverman, 2003; Skelly and Werner, 1990). Although some tadpoles have been shown to be able to mitigate the intrinsic costs of induced defenses by adjusting their metabolic rate (Azevedo-Ramos et al., 1992), the trade-off between defense behavior and the cost of reduced activity are likely to constitute a main driver in the evolution of anti-predator responses in tadpoles.

Despite studies showing various kinds of anti-predator behaviors in tadpoles and demonstrating the adaptive benefits and trade-offs of these behaviors, little is known about how tadpoles detect and avoid predators. Several studies have shown that larval amphibians respond to chemical cues, either coming directly from potential predators (kairomones, e.g. Petranka, 1989; Feminella and Hawkins, 1994; Kats and Dill, 1998) or resulting from predation on conspecifics (alarm substances, e.g. blood; Maag et al., 2012; see also Schoeppner and Relyea, 2009). Visual cues are generally assumed to be insufficient for precise predator discrimination, as amphibian larvae are typically myopic (Mathis and Vincent, 2000). However, to date there is only limited knowledge about the relevance of visual cues for predator detection in tadpoles, or the cross-modal integration from different sensory inputs (but see Hettyey et al., 2012; Stauffer and Semlitsch, 1993; Stynoski and Noble, 2011; Takahara and Yamaoka, 2009).

In the present study, we investigated anti-predator behavior in tadpoles of the Neotropical brilliant-thighed poison frog *Allobates femoralis* (Boulenger, 1884) (Dendrobatidae: Aromobatinae *sensu* AmphibiaWeb, 2021), a species that occurs throughout the Amazon basin and the Guyana shield in disjunctive local populations (Amézquita et al., 2009). During the reproductive season, males call from slightly elevated structures on the forest floor to announce territory possession to male competitors (Kaefer et al., 2012) and to attract females (Roithmair, 1992; Ringler et al., 2009). Both sexes are iteroparous and polygamous (Ursprung et al., 2011) within prolonged but discrete reproductive periods that coincide with the local rainy seasons (Gottsberger and Gruber, 2004). Pair formation, courtship and mating take place in the male's territory (Montanarin et al., 2011; Stückler et al., 2019; Fischer et al., 2020), where externally fertilized terrestrial clutches of approximately 20 eggs are laid in the leaf litter (Weygoldt, 1980). Tadpole transport takes place after 15–20 days of larval development and is mainly performed by males (Ringler et al., 2013) but females take over when the male disappears (Ringler et al., 2015b). Males are able to assess and differentiate between aquatic predators (McKeon and Summers, 2013), and allocate tadpoles across several water bodies, probably as a bet-hedging strategy against total offspring loss (Erich et al., 2015; Ringler et al., 2018). Tadpoles are non-cannibalistic (but may consume dead conspecifics; E.R., personal observation), require 40–50 days until metamorphosis, and reach sexual maturity after 8 (males) to 10 months (females) (Weygoldt, 1980). Common predators of *A. femoralis* tadpoles are dragonfly larvae (Odonata), spiders, snakes and heterospecific carnivorous tadpoles, for example of the dyeing poison frog (*Dendrobates tinctorius*; Dendrobatidae: Dendrobatinae; R.M., A.P., M. Ringler and E.R., personal observation).

The aims of this study were: (1) to investigate differences in anti-predator behavior, specifically tadpole movement strategies, in response to different predators; and (2) to identify whether chemical, visual or both types of cues are used in predator detection and discrimination.

We hypothesized that tadpoles may benefit from exhibiting limited movement when exposed to a predator but may show differential avoidance behavior depending on the predator’s foraging mode (i.e. ambush/sit-and-wait strategy seen in some odonate larvae or active foraging seen in some other odonate larvae and *D. tinctorius* tadpoles; Johansson, 1991; Fouilloux et al., 2020 preprint; Pritchard, 1965; Rojas, 2014). We also hypothesized that the use of multiple cues may improve predator recognition and avoidance.

Accordingly, we predicted that: (1) tadpoles would show reduced movement in predator trials when presented with predator cues compared with control trials with no predator stimuli; (2) tadpoles would differ in the information (chemical, visual or both) used to detect certain predators and in the type of response, possibly related to the predator’s foraging mode; and (3) tadpoles would show stronger anti-predator responses in the presence of both visual and chemical predator stimuli, compared with uni-modal treatments.

## MATERIALS AND METHODS

Experiments were performed in spring 2013 (21 January to 15 March 2013) in a lowland rainforest near the field camp ‘Saut Pararé’ (4°02′N, 52°41′W) of the CNRS Nouragues Ecological Research Station in the nature reserve Les Nouragues, French Guiana (Bongers et al., 2001). This study was approved by the scientific committee of the Nouragues Ecological Research Station. All sampling was conducted in strict accordance with current French and EU law and followed the ASAB guidelines (ASAB, 2020).

**Fig. 1.**
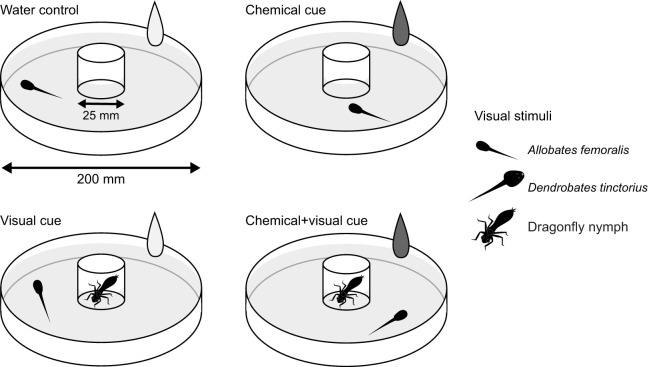
**Experimental setup.** Four identical arenas (200 mm diameter) were used in each trial and therefore four tadpoles were tested at the same time, one in each stimulus group. Water depth in arenas was 20 mm. In the water control, the central cylinder (25 mm diameter) was empty and 50 ml of tap water was added to the arena. In the olfactory (chemical) cue group, the central cylinder was empty and 50 ml of stimulus solution (from a conspecific *Allobates* *femoralis* tadpole, a heterospecific *Dendrobates* *tinctorius* tadpole or an odonate dragonfly larvae) was added. In the visual cue group, the central cylinder housed a stimulus individual (a conspecific *A. femoralis* tadpole, a heterospecific *D. tinctorius* tadpole or an odonate dragonfly larvae) and 50 ml of tap water was added to account for water movement. In the chemical+visual cue group, the central cylinder housed a stimulus individual and 50 ml of stimulus solution was added to the arena.

We designed an experiment where we placed tadpoles inside a round arena (opaque, white resin bowl, 100 mm radius) with a grid on the bottom. In the center of each arena, we mounted a glass cylinder with a diameter of 25 mm that extended over the water surface in the bowl, where we could display visual cues. This glass cylinder was present in all trials, regardless of the use of a visual cue. We exposed test tadpoles to one of three comparative treatments: (1) conspecific, non-threatening tadpoles (*A. femoralis*), hereinafter referred to as the ‘femoralis’ treatment (*N*=64 focal tadpoles, *N*=16 per stimulus group), (2) heterospecific, predatory tadpoles (*D. tinctorius*), hereafter the ‘tinctorius’ treatment (*N*=76 focal tadpoles, *N*=19 per stimulus group), or (3) predatory dragonfly larvae (*Aeshna* sp.), the ‘dragonfly’ treatment (*N*=64 focal tadpoles, *N*=16 per stimulus group). These treatments were all presented in the following setup (stimulus groups): one arena contained pure tap water (untreated water from a natural well close to the river; control), the second presented only olfactory (chemical) cues of the presented species used in the respective treatment, the third provided only the visual cue of the respective treatment species, and the fourth arena provided both visual and chemical cues (Fig. 1). This setup allowed us to simultaneously test four tadpoles.

We collected *A. femoralis* tadpoles *ad libitum* from artificial water bodies that had been installed in the course of a previous study (Ringler et al., 2015a). We only took tadpoles from pools where no predators were present; thus, we assumed them to be naive regarding experience with predators. We only tested tadpoles between Gosner stages 26 and 30 (Gosner, 1960) to ensure similar developmental stages across tadpoles. Therefore, we were unable to use a within-individual test design. Before testing, we photographed each tadpole on scale paper to obtain standardized pictures for later size measurements. Larval body size was calculated as the length from the tip of the mouth to the onset of the tail, using the program ImageJ (http://imagej.nih.gov/ij/, accessed 7 January 2020).

Before each trial, we thoroughly rinsed the arenas and filled them with 500 ml of fresh tap water to a depth of 20 mm. After placing a tadpole inside an arena, we allowed it to acclimate to the testing environment for 15 min. Then, we added the visual and chemical cues to the respective arenas and recorded the subsequent movement of test tadpoles using digital video cameras (SDR-SW20, Panasonic, Kadoma, Japan; 576×704 pixels, 25 frames s^−1^). Filming started before tadpoles and cues were added and lasted at least 15 min without any further intervention and the four arenas were filmed simultaneously. We performed trials between 11:00 h and 17:00 h. An opaque tarpaulin was positioned just above the setup to avoid strong reflectance on the water surface and shadow casting by the experimenters.

To obtain the stimulus solution containing the chemical cues, we used bowls identical to the trial arenas and placed a conspecific (*A. femoralis*) or heterospecific (*D. tinctorius*) tadpole, or a dragonfly larva in water for at least 48 h without adding food. By using this method of keeping predators without food, we were able to obtain predator kairomones only. Larval dragonflies and *D. tinctorius* tadpoles were collected from pools located in the study area and kept in small containers filled with tap water. We made sure that all predators were of similar size to control for possible effects of predator size on focal tadpole behavior. Multiple predator individuals were used to allow them to take up food during the testing period, as they were collected from natural water bodies in the study area, but keep them starved for 48 h before use to exclude interference from cues produced by predation. The *A. femoralis* tadpoles that were kept in a container for collecting the chemical ‘femoralis’ cues were never used as test subjects in any of the trials. For the presentation of chemical cues, we added 50 ml of the treated water to the trial areas by pouring it in one continuous movement around the central glass cylinder. Trials without chemical cues (i.e. control and visual) received an identical volume of water, to ensure the same handling effects across all trials.

We provided visual cues by placing glass cylinders containing the stimulus individual (femoralis, tinctorius or dragonfly larvae) in the middle of the arena. In trials without visual cues, an empty glass cylinder was simultaneously placed to standardize handling. In the bimodal condition (chemical+visual) we used the visual cue (introduced first) as well as the stimulus solution (‘treated’ water poured in immediately after the visual cues were added) to present the two cues simultaneously. All trials were always conducted by two experimenters to ensure equal handling and starting time for all trials in one set.

We tested each tadpole only once (between-individual test design) and placed it in a separate tank after completion of the trial to prevent pseudo-replication due to eventual repeated use of single tadpoles. After the experiment was completed, we released all tadpoles across pools inside the study area where they were collected.

### Data extraction

Tadpole movements were analyzed by tracking individual movement patterns with a custom-made ‘Tadpole Video Analysis’ application (available upon request from J.O.). The program is coded in Python 2.7 (https://www.python.org/; accessed 7 March 2021) and uses the external libraries NumPy (van der Walt et al., 2011), OpenCV (Bradski and Kaehler, 2000) and wxPython (https://www.wxpython.org/). The tracking of tadpole movements was started immediately after the addition of the stimuli, as soon as the water surface was calm (<1 s). After the locations of tadpoles across all frames had been determined (for more details, see Supplementary Materials and Methods), the program calculated the distance to the respective center of the bowls (i.e. the location of the dragonfly larvae, heterospecifics or conspecifics) and distance traveled from the previous frame by using the known size of the bowl (100 mm radius). Because we used an automated tracking program, we can exclude observer bias influencing our measurements. We only analyzed the first 3 min (4500 frames) of each trial to capture the immediate response of the focal tadpole and to avoid habituation to the presented stimuli over time.

### Statistical analyses

#### Active predator avoidance

We investigated whether the tadpoles were attracted to or avoided the center of the arena by looking at the change in the cumulative distance moved towards/away from the center across frames after stimulus presentation. First, we made sure that the starting position of tadpoles across stimulus groups did not differ, by comparing the position of tadpoles at frame 1 across stimulus groups using a Kruskal–Wallis test. Then, we calculated the distance moved by each tadpole towards/away from the center from one frame to the next, and calculated the cumulative sum of these measurements for our statistical analysis. If tadpoles were attracted to the center by a stimulus, the cumulative sum would decrease across frames; if tadpoles avoided the center, the cumulative sum would increase; no reaction would show as no change over frames.

We used the cumulative sum moved towards/away from the center as the response variable in a linear mixed effects model (LME, R package lmerTEST; Kuznetsova et al., 2017) with type of stimulus used (stimulus group: water control, chemical, visual, chemical+visual), time of day a tadpole was tested (noon and afternoon – before or after 14:00 h) and the interaction of stimulus group with frame as the fixed effects to account for swimming behavior across time. We made sure to order the factor levels of stimulus group to include the water control in the intercept (first level) to allow a comparison of behavior in the groups (chemical, visual and chemical+visual) with the water control. The data from all control individuals regardless of treatment (femoralis, tinctorius and dragonfly) were used for comparison in all analyses. We included a random intercept (tadpole ID) and random slope (frame) to account for repeated measures across frames. Frame was scaled and centered for better model performance. We visually inspected Q–Q plots to ensure that model residuals conformed to the assumption of normality and transformed response variables if this assumption was not met (see provided R script for details: doi:10.17605/OSF.IO/ESRYN). To be able to apply data transformation, we shifted the whole dataset upwards by adding the absolute maximum value to all values. By doing so, we were able to remove negative values but kept the relative movement behavior of the tadpoles towards/away from the center. We ran one model for each treatment group (femoralis, tinctorius and dragonfly). To ensure that our results were robust and not an artefact of the time frame chosen for analysis, we ran these models across 1500 (1 min), 3000 (2 min) and 4500 frames (3 min). Furthermore, we ran an additional analysis without animals that started within 20 mm of the arena edge to ensure that our results were not affected by starting position.

Finally, we also compared the cumulative sum moved towards/away from the center at frame 1500 (1 min), 3000 (2 min) and 4500 (3 min) across stimulus groups (water control, chemical, visual, chemical+visual) using a Kruskal–Wallis test to see whether the tadpoles' behavior persisted across frames.

#### Reduced activity (freezing behavior)

We investigated whether the tadpoles reduced activity as a response to the stimuli presented by looking at the cumulative distance traveled (cumulative sum of the distance moved from frame to frame) across frames. We applied LME models with the cumulative distance traveled as the response variable, and stimulus group (water control, chemical, visual, chemical+visual), frame and time of day tested as well as the interactions between stimulus group and time of day with frame as the fixed effects. Again, factor levels were ordered so as to ensure that the control group was included in the intercept. The data from all control individuals regardless of treatment (femoralis, tinctorius and dragonfly) were used for comparison in all analyses. We included a random intercept (tadpole ID) and random slope (frame) to account for repeated measures across frames. Frame was scaled and centered for better model performance. We visually inspected Q–Q plots to ensure that model residuals conformed to the assumption of normality and transformed response variables if this assumption was not met (see provided R script for details: doi:10.17605/OSF.IO/ESRYN). We ran one model for each treatment group (femoralis, tinctorius and dragonfly). To ensure that our results were robust and not an artefact of the time frame chosen for analysis, we ran these models across 1500 (1 min), 3000 (2 min) and 4500 frames (3 min).

Again, we compared the cumulative distance traveled at frame 1500 (1 min), 3000 (2 min) and 4500 (3 min) across stimulus groups (water control, chemical, visual, chemical+visual) using a Kruskal–Wallis test to see whether the tadpoles' behavior persisted across frames.

#### Tadpole size

We were unable to control for exact tadpole size during testing and ran an analysis to find out whether tadpole size was correlated with the cumulative distance moved towards/away from the center and distance traveled. To exclude effects of stimulus group on behavior (designed to induce anti-predator behavior), we only analyzed behavior of individuals from the water control across the three treatment groups (femoralis, tinctorius and dragonfly). We ran a LME model with size as the response variable and cumulative distance moved towards/away from the center or cumulative distance traveled as the fixed effects. To account for differences in behavior resulting from being tested in different treatment groups (femoralis, tinctorius and dragonfly), we included treatment as a random effect. We ensured that model assumptions were met by visually inspecting plots.

All statistical analyses were run in R version 4.0.3 (http://www.R-project.org/). All tests were two tailed and α was set to 0.05. All datasets generated and the code used for analyses in the current study are available at the Open Science Framework (doi:10.17605/OSF.IO/ESRYN).

## RESULTS

### Active predator avoidance

**Fig. 2.**
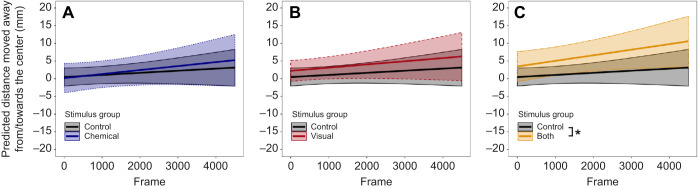
**Predicted changes in the cumulative distance moved towards/away from the center across frames in the dragonfly treatment group.** (A) Comparison between the water control group (*N*=51) and the chemical group (*N*=19). (B) Comparison between the water control group (*N*=51) and the visual group (*N*=19). (C) Comparison between the water control group (*N*=51) and the multi-modal (chemical+visual) group (*N*=19). Differences were analyzed using linear mixed effects models (LME). The intercept of the multi-modal group significantly differed from that of the water control group (LME, 4500 frames, estimate=0.135, lower–upper confidence interval (CI)=0.019–0.250, *t*=2.242, *P*=0.028; Table S1) and so did the change across frames (LME, 1500 frames, estimate=0.051, CI=0.009–0.094, *t*=2.339, *P*=0.031; Table S1). *P<0.05.

Tadpoles tested in the different stimulus groups started a trial from similar positions (Kruskal–Wallis test, χ^2^=0.908, d.f.=3, *P*=0.824). Tadpoles neither avoided nor were attracted to the center regardless of stimulus modality in both the femoralis and tinctorius treatment (LME, *P*>0.05; Table S1, Fig. S1). Tadpoles avoided the center of the arena when a combination of both chemical and visual cues originating from dragonfly larvae was added [LME, estimate=0.135, lower–upper confidence interval (CI)=0.019–0.250, *t*=2.242, *P*=0.028; Fig. 2C]. This effect was, however, only detectable when 3000 or 4500 frames were included in the analysis, indicating that it was quite weak (Table S1). Interestingly, when looking at the first 1500 frames only, tadpoles significantly increased the distance to the center in the multi-modal treatment, indicated by a significant interaction between stimulus group and frame (LME, estimate=0.051, CI=0.009–0.094, *t*=2.339, *P*=0.022). This effect became non-significant with the inclusion of 3000 and 4500 frames, showing that the reaction was short lived (Table S1). The analysis only including those individuals that were within 80 mm of the center showed the same effects (Table S1). Furthermore, we found no difference in the cumulative distance moved towards/away from the center after 1500 frames (1 min), further indicating that tadpoles' reaction to the stimuli was immediate but short lived (Kruskal–Wallis test, χ^2^=4.645, d.f.=3, *P*=0.200; Table S2). We found no difference in behavior when tadpoles were tested at noon or in the afternoon (LME, *P*>0.05; Table S1). Overall, tadpole behavior was consistent across frames (Kruskal–Wallis test, *P*>0.05; Table S2).

### Reduced activity (freezing behavior)

**Fig. 3.**
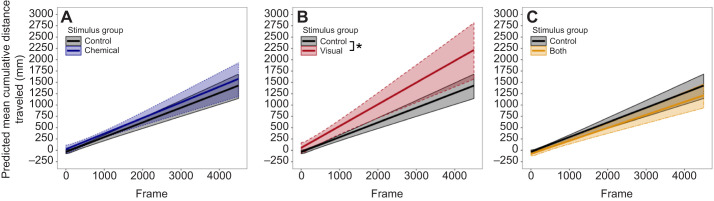
**Predicted changes in the cumulative distance traveled across frames in the femoralis treatment group.** (A) Comparison between the water control group (*N*=51) and the chemical group (*N*=16). (B) Comparison between the water control group (*N*=51) and the visual group (*N*=16). (C) Comparison between the water control group (*N*=51) and the multi-modal (chemical+visual) group (*N*=16). Differences were analyzed using LME. The intercept of the visual group significantly differed from that of the water control group (LME, 4500 frames, estimate=7.775, CI=3.233–12.317, *t*=3.296, *P*=0.001; Table S1) and so did the change across frames (LME, 1500 frames, estimate=1.678, CI=0.395–2.962, *t*=2.517, *P*=0.037; Table S1). *P<0.05.

We found that tadpoles moved more after a conspecific was introduced into the central cylinder (visual stimulus group; LME, estimate=7.775, CI=3.233–12.317, *t*=3.296, *P*=0.001; Fig. 3B). Tadpoles also increased movement across frames (LME, estimate=1.678, CI=0.395–2.962, *t*=2.517, *P*=0.037; Fig. 3B) but this was only significant when 1500 frames were analyzed (Table S1). This increase in movement was still detectable after 1500 frames (Kruskal–Wallis test, χ^2^=13, d.f.=3, *P*=0.005) and 3000 frames (Kruskal–Wallis test, χ^2^=9.018, d.f.=3, *P*=0.029) but not after 4500 frames (Kruskal–Wallis test, χ^2^=6.781, d.f.=3, *P*=0.079). Tadpoles neither increased nor decreased movement when stimuli from a dragonfly or heterospecific predatory tadpole were introduced into the arena (Table S1, Fig. S1). In both predator treatments (dragonfly and tinctorius), tadpoles moved less when tested later in the day (LME, *P*>0.05; Table S1) and this effect was detectable when 3000 and 4500 frames but not 1500 frames were analyzed (LME, *P*<0.05; Table S1). Overall, tadpole behavior was consistent across frames (Kruskal–Wallis test, *P*>0.05; Table S2).

### Tadpole size

The analysis looking at a possible correlation between tadpole size and tadpole behavior (cumulative distance moved towards/away from the center and cumulative distance traveled) showed that, within the water control, size did not correlate with cumulative distance moved towards/away from the center (LMER, estimate=−0.0014, CI=−0.0036–0.0007, *t*=−1.312, *P*=0.197) or distance traveled (LMER, estimate=−0.00002, CI=−0.00005–0.00001, *t*=−1.184, *P*=0.244) indicating that behavior of individuals between Gosner stages 26 and 30 (Gosner, 1960) does not innately differ.

## DISCUSSION

Our results demonstrate that *A. femoralis* tadpoles show active avoidance of a predator stimulus when the stimulus originates from a dragonfly larva. Tadpoles either moved away from the center or remained at the edge of the arena throughout the experiment. Importantly, this avoidance behavior only occurred when both visual and olfactory information were available, was immediate and decreased over time. No changes in behavior occurred when stimuli originated from a conspecific or predatory heterospecific tadpole. We also show that focal tadpoles increased swimming activity when visual information from a conspecific was presented but did not change activity in response to stimuli from predators. Interestingly, our results demonstrate that tadpole activity decreased in the afternoon in both predator treatments but not in the conspecific femoralis treatment.

We had three predictions regarding the reaction of our focal tadpoles to the different predatory cues provided in our experiment. First, we predicted that tadpoles would show reduced movement when presented with predator stimuli compared with control trials with no predator stimuli. Our results do not support this hypothesis as tadpoles did not decrease swimming activity in response to predator kairomones. Studies investigating anti-predator behavior in anuran tadpoles often use a combination of kairomones and conspecific alarm cues, or feed predators with prey (e.g. Mandrillon, 2005; Schoeppner and Relyea, 2005; Mirza et al., 2006; Lucon-Xiccato et al., 2016; Supekar and Gramapurohit, 2020). In our experiments, we only used kairomones from starved predators, which might have resulted in an overall weaker response in our focal tadpoles. Tadpoles of *Indosylvirana temporalis* did show anti-predator behavior (stayed away from the predator, reduced distance traveled and increased burst speed) in response to chemical cues from tadpole-fed predators but not in response to starved predators. Furthermore, experienced *I. temporalis* tadpoles showed stronger responses compared with naive individuals (Mogali et al., 2012). This could explain why our tadpoles did not reduce traveling distance by becoming immobile in response to any predator stimuli. Such a response might only occur when conspecific alarm cues or cues from prey fed predators are present as an indicator of immediate danger. Furthermore, animals might show different behaviors or levels of response to predator kairomones, alarm cues and a combination of the two (Schoeppner and Relyea, 2005).

Second, we predicted that tadpoles would differ in the information (chemical, visual or both) used to detect certain predators. We are unable to confirm or refute this hypothesis because we were able to detect a response in our focal tadpoles to predator stimuli from dragonfly larvae but not to predatory, heterospecific *D. tinctorius* tadpoles. Either naive *A. femoralis* tadpoles are unable to discriminate between conspecific and heterospecific tadpoles or they do not perceive them as a threat. Our focal tadpoles did increase swimming activity in response to visual cues from conspecifics but not in response to heterospecifics. Furthermore, such an increased activity was not shown in the bi-modal condition in which both visual and chemical cues were available. This indicates that this result might be an artefact of our between-individual test design. Nonetheless, this results could indicate that *A. femoralis* tadpoles can discriminate conspecifics from heterospecifics but do not perceive them as a threat and, possibly, need to gain experience to show a response. Previous work demonstrated that animals can recognize and distinguish between different predators (e.g. Feminella and Hawkins, 1994; Hettyey et al., 2011; Kiesecker et al., 1996; Supekar and Gramapurohit, 2018), use different modalities to recognize them (e.g. Kiesecker et al., 1996) and show distinct behavioral responses to these different predators (e.g. Schmidt and Amézquita, 2001). *Allobates femoralis* tadpoles might not innately recognize all predators but need to learn to respond to certain predator species (e.g. Crane et al., 2017; Epp and Gabor, 2008; Gonzalo et al., 2009).

We also predicted that tadpoles would respond differently depending on the predator type, as a result of differences in foraging mode. Our data do not support a differential response because tadpoles did not show any reaction to the heterospecific *D. tinctorius* tadpoles. However, our results indicate a stronger reaction (less swimming activity) to predator stimuli later in the day, which could be associated with increased predator activity later in the day. Our focal tadpoles moved less in the afternoon in both the dragonfly and tinctorius treatment. Previous work has demonstrated that temporal variation in predation risk can affect the intensity of anti-predator responses, which was formulated under the ‘risk allocation hypothesis’ (RAH; Lima and Bednekoff, 1999). For example, red-backed salamanders (*Plethodon cinereus*) show stronger anti-predator behavior to chemical cues originating from garter snakes (*Thamnophis sirtalis*) earlier at night, probably because garter snakes are more active earlier at night (Sullivan et al., 2005). Similarly, tadpoles of the wood frog (*Lithobates sylvaticus*) trained to expect predator odor (from a tiger salamander, *Ambystoma tigrinum*) during the mornings, later responded more strongly to that odor in the mornings than they did at night. A second group that was treated in the opposite way (receiving the predator odor in the evening) showed a stronger response in the evenings (Ferrari et al., 2008; Ferrari and Chivers, 2009). We currently have no knowledge about the activity pattern of the predator species we used. Another possibility is that lower water temperature in the afternoon could have reduced swimming activity in the tadpoles. However, we can safely exclude this alternative, because temperatures generally increase across the day and we found no decrease in activity during the afternoon in the femoralis group. Nonetheless, future studies should record water temperature to exclude such effects.

Lastly, we predicted that tadpoles would show stronger anti-predator responses in the presence of both visual and chemical cues, compared with uni-modal cues. Our results confirm this hypothesis as we found that *A. femoralis* tadpoles avoided the center of an arena in the dragonfly treatment only if both visual and chemical information was presented. The presence of mere chemical cues alone could provide information about the presence of a predator but not provide sufficient information on the appropriate escape response such as directionality of escape. Visual cues alone might not suffice to elicit a response because visibility might be low as a result of the prey's low visual acuity or, in aquatic ecosystems, high water turbidity. Active avoidance of predator stimuli together with decreased activity was shown in common frog (*Rana temporaria*) and *I. temporalis* tadpoles when presented with a caged dragonfly nymph (Laurila et al., 1997; Mogali et al., 2012), water including dragonfly kairomones (Laurila, 2000) or water from prey-fed predators (Mogali et al., 2012). From our current results, it is not clear whether uni-modal cues are used by *A. femoralis* tadpoles to detect predators. Effects of perturbation at the start of a trial might have masked weaker responses in the uni-modal groups, preventing us from reliably detecting them. Tadpoles of *A. femoralis* might, however, need both visual and chemical information to show a defense reaction. Whether uni-modal cues can be used by our tadpoles should be investigated in a future experiment controlling for perturbation at the start of a trial.

Importantly, to our knowledge, this is the first study investigating tadpole anti-predator responses in a poison frog (Dendrobatidae *sensu* AmphibiaWeb, 2021) with terrestrial egg deposition and tadpole transport to small terrestrial pools (but see Stynoski and Noble, 2011). In this frog family, terrestrial egg deposition with tadpole transport to water bodies is consider the synapomorphic breeding mode (Lötters et al., 2007; Crump, 2015; Grant et al., 2017). In our study species, eggs are externally fertilized and laid in the leaf litter (Weygoldt, 1980). After 15–20 days of larval development, the tadpoles are transported and spread across several water bodies mainly by males (Ringler et al., 2013). Males base their decisions on where to deposit tadpoles on pool size but also on the presence or absence of predators (McKeon and Summers, 2013; see also Correa and Rodrigues, 2015; Brown et al., 2008 and Schulte et al., 2013 for similar findings in other anuran and dendrobatid species). Consequently, selection to evolve anti-predator measures might be weak in *A. femoralis* tadpoles. Parental care could be the most effective anti-predator measure in our study system because fleeing or freezing behavior to avoid predators might only be of limited success in small water bodies.

The response shown by the tadpoles in this study was probably innate recognition. Tadpoles were collected from pools without visible predators and are unlikely to have had experience with the predators used in our study. Future studies could look at how tadpoles behave in response to predator stimuli when they have prior experience with predation. Such a setup would also allow the investigation of how conspecific alarm substances affect tadpole behavior and whether learning is involved in predator recognition in *A. femoralis*.

In summary, contrary to our expectations and previous studies in other anuran species (Batabyal et al., 2014; Petranka and Hayes, 1998; Takahara et al., 2012; but see Hettyey et al., 2011), we found no reduction in activity (distance traveled across frames) in *A. femoralis* tadpoles but instead an increase in swimming activity in response to visual cues of a conspecific. Our data demonstrate, however, that focal tadpoles did actively avoid the source of predator stimuli by staying further away from the center of the arena when both visual and chemical information from a dragonfly larva was present. Finally, our study shows that tadpoles of the poison frog *A. femoralis* rely on multimodal cues to detect certain predators but could require previous experience to recognize less-common predators. This is, to our knowledge, the first study looking at anti-predator behavior in tadpoles of a poison frog, providing a stepping stone to further investigations that might elucidate whether and how conspecific alarm substances alter tadpole anti-predator behavior.

## Supplementary Material

Supplementary information
