# Supplementary material for: Naive poison frog tadpoles use bi-modal cues to avoid insect predators but not heterospecific predatory tadpoles
Source: J Exp Biol. 2021 Dec 16;224(24):jeb243647. doi: 10.1242/jeb.243647 (PMC8729909; doi:10.1242/jeb.243647)
Supplement: Supplementary information [file jexbio-224-243647-s1.pdf]

Supplementary Materials and Methods

Tadpole Video Analysis

The program is semi-automatic: behaviour is coded automatically but a human supervisor can provide user input. The algorithm processes the video data frame by frame and determines the positions of tadpoles in the four bowls by colour tracking. Before running the script, a few manual adjustments had to be made. First, we defined the centres of all four arenas via mouse clicks onto the first image in the video. The program than automatically assigned a circle of 100 mm radius around each of these points representing the four areas, where all subsequent measurements were taken. Second, we defined the centre of the tadpole body, by drawing a circle around the tadpole body, to set the initial search areas for the tracking algorithm. The colour tracking was conducted using OpenCV’s inRange function with minimum and maximum HSV (Hue, Saturation, Value) parameters. Hue and Saturation parameters were less relevant due to the tadpole’s dark grey colour. The Value parameter was the important parameter to detect the tadpole. However, the Value of tadpole was not always consistent throughout the video depending on the position of the tadpole and lighting condition changes in outdoor experimental setup. Therefore, the maximum Value parameter for detecting tadpole in each frame was automatically adjusted using a median pixel value in a small area around the last known tadpole position. The small area is a two pixels wide circular area, which is eight pixels away from the last tadpole position. With this automatic adjustment, the range of target (tadpole) colour is changed (darker or lighter) according to overall lighting intensity around the tadpole. After the target colour range adjustment, the colour tracking was applied in the 10-pixel-radius area centred at the last tadpole position to determine the tadpole position in the current frame. In general, this automatic adjustment functioned as intended. However, if the program could not successfully track the tadpole, which was indicated by the absence of a coloured dot on top of the tadpole, the observer could manually determine the tadpole position for a given frame by clicking the left mouse button and draw a circle around the tadpole body to reset the search area for the tracking algorithm. Subsequently the automatic tracking algorithm was resumed.

Journal of Experimental Biology • Supplementary information

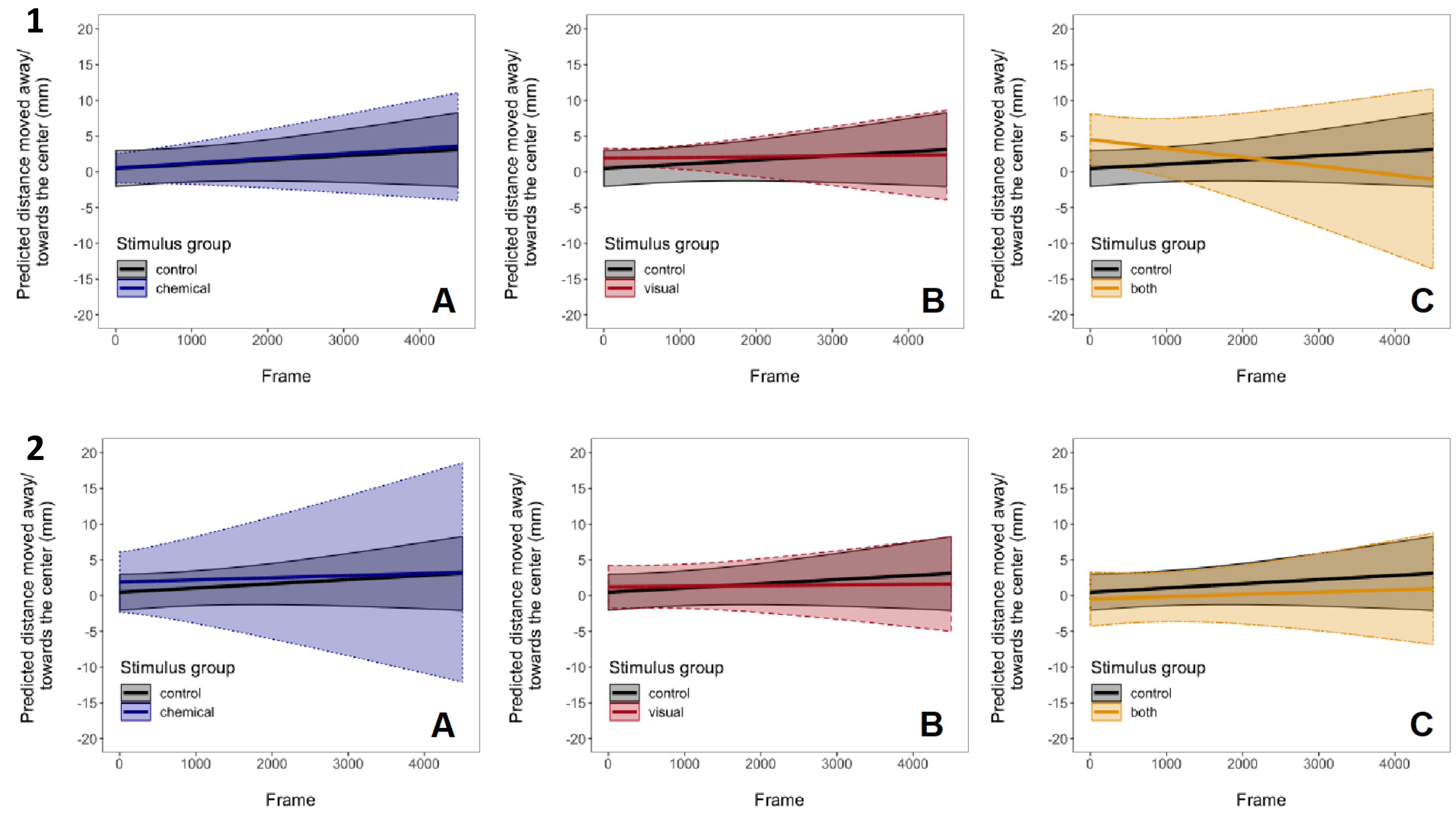

Journal of Experimental Biology • Supplementary information

3

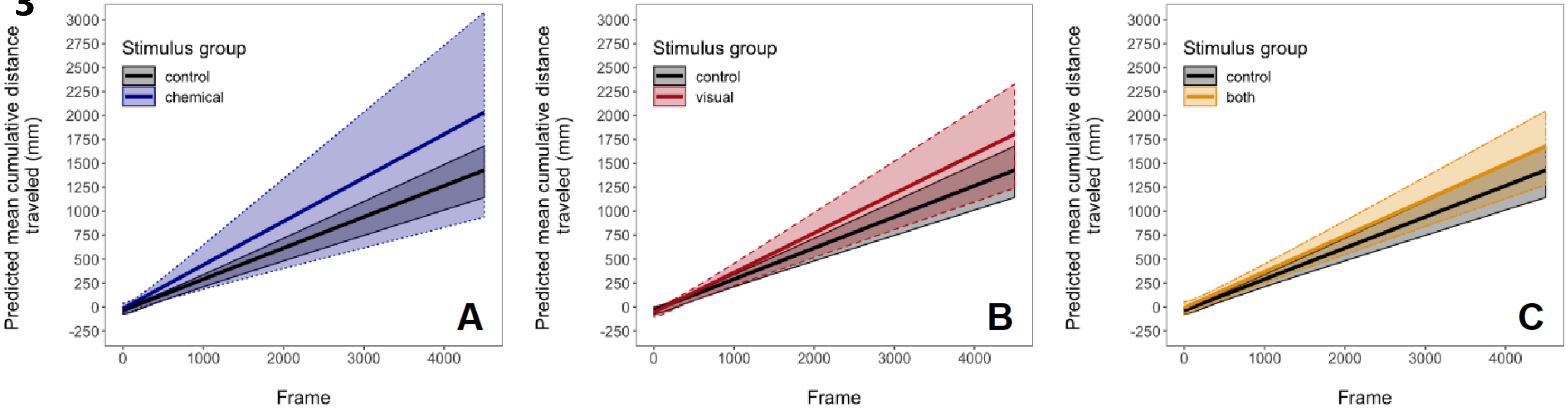
Predicted mean cumulative distance traveled (mm)
Frame
Stimulus group
control
chemical
visual
both
A
B
C

4

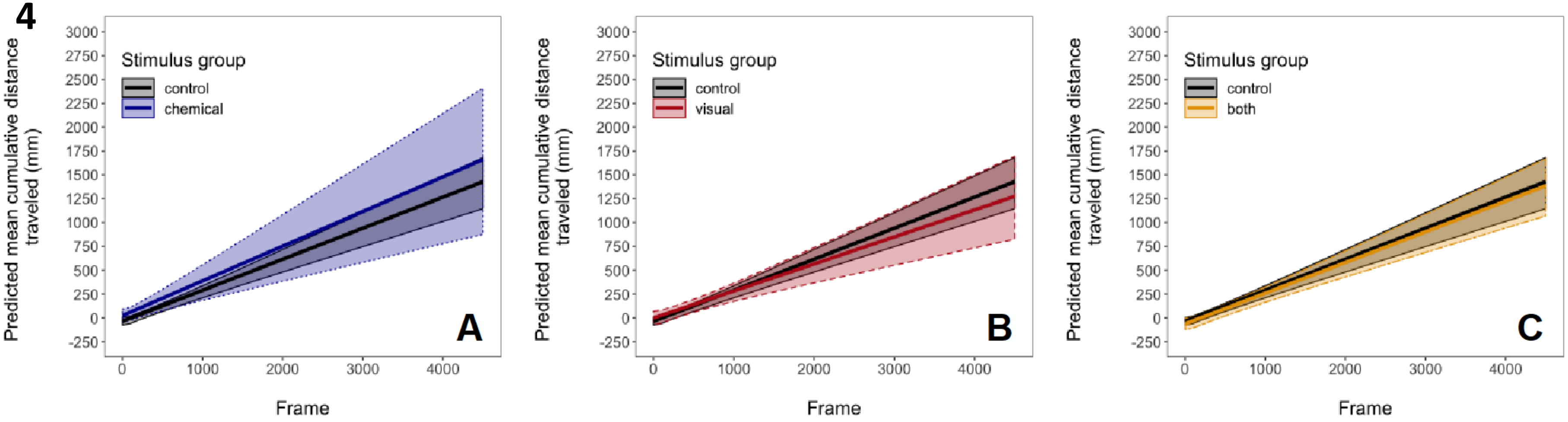
Predicted mean cumulative distance traveled (mm)
Frame
Stimulus group
control
chemical
visual
both
A
B
C

Fig. S1. Predicted changes in tadpole movement across frames. Panel ONE: The cumulative distance moved away/ towards the center (mm) by individuals in the femoralis treatment group. A) Comparison between the water control group (N = 51; black, solid line) and the chemical group (N = 16; blue, dotted line). B) Comparison between the water control group (N = 51; black, solid line) and the visual group (N = 16; red, dashed line). C) Comparison between the water control group (N = 51; black, solid line) and the multi-modal (both chemical and visual) group (N = 16; yellow, double dashed line). Panel TWO: The cumulative distance moved away/ towards the center (mm) by individuals in the tinctorius treatment group. A) Comparison between the water control group (N = 51; black, solid line) and the chemical group (N = 16; blue, dotted line). B) Comparison between the water control group (N = 51; black, solid line) and the visual group (N = 16; red, dashed line). C) Comparison between the water control group (N = 51; black, solid line) and the multi-modal (both chemical and visual) group (N = 16; yellow, double dashed line). Panel THREE: The cumulative distance travelled (mm) by individuals in the dragonfly treatment group. A) Comparison between the water control group (N = 51; black, solid line) and the chemical group (N = 19; blue, dotted line). B) Comparison between the water control group (N = 51; black, solid line) and the visual group (N = 19; red, dashed line). C) Comparison between the water control group (N = 51; black, solid line) and the multi-modal (both chemical and visual) group (N = 19; yellow, double dashed line). Panel FOUR: The cumulative distance travelled (mm) by individuals in the tinctorius treatment group. A) Comparison between the water control group (N = 51; black, solid line) and the chemical group (N = 16; blue, dotted line). B) Comparison between the water control group (N = 51; black, solid line) and the visual group (N = 16; red, dashed line). C) Comparison between the water control group (N = 51; black, solid line) and the multi-modal (both chemical and visual) group (N = 16; yellow, double dashed line). Differences were analyzed using linear mixed models.

Table S1. Parameter estimates and test statistics resulting from the model investigating difference in distance moved towards or away from the centre and cumulative distance travelled between stimulus groups (control = intercept, chemical, visual, chemical & visual) across 1500 frames (1 minute), 3000 frames (2 minutes) and 4500 frames (3 minutes). Time of day (noon or afternoon) was also included in the model as a fixed effect. The response variable in models analysing distance moved towards or away from the centre was cube root transformed. In models analysing cumulative distance travelled, we square root transformed the response variable. The model included a random intercept (tadpole ID) and slope (frame) and frame was scaled and centred for better model performance. Significant ($p < 0.05$) parameters are highlighted in bold. CI – confidence interval

DISTANCE MOVED TOWARDS OR AWAY FROM THE CENTER							
Femoralis treatment all individuals							
		**Parameter**	**Estimate**	**Lower CI**	**Upper CI**	***t*-value**	***p*-value**
1500 frames	Main effect	**Intercept**	**4.217**	**4.176**	**4.257**	**199.963**	**< 2*10^{-16}**
		Frame	-0.003	-0.022	0.016	-0.349	0.728
		Chemical	-0.006	-0.075	0.063	-0.169	0.866
		Visual	0.002	-0.067	0.071	0.042	0.967
		Chemical & visual	0.029	-0.040	0.098	0.807	0.422
		Noon	-0.023	-0.067	0.021	-1.035	0.304
	Interaction	Frame – chemical	0.023	-0.014	0.062	1.222	0.225
		Frame – visual	0.024	-0.014	0.061	1.207	0.231
		Frame – chemical & visual	0.020	-0.017	0.058	1.045	0.299
3000 frames	Main effect	**Intercept**	**4.210**	**4.155**	**4.264**	**148.954**	**< 2*10^{-16}**
		Frame	-0.026	-0.060	0.009	-1.456	0.149
		Chemical	0.011	-0.086	0.108	0.218	0.828
		Visual	0.029	-0.068	0.127	0.584	0.561
		Chemical & visual	0.024	-0.073	0.121	0.478	0.634

Journal of Experimental Biology • Supplementary information

		Noon	-0.046	-0.096	0.004	-1.778	0.079
	Interaction	Frame – chemical	0.036	-0.033	0.105	1.014	0.314
		Frame – visual	0.044	-0.025	0.113	1.236	0.220
		Frame – chemical & visual	0.003	-0.066	0.072	0.083	0.934
4500 frames	Main effect	**Intercept**	**4.226**	**4.163**	**4.290**	**128.362**	**< 2*10^{-16}**
		Frame	0.006	-0.026	0.038	0.382	0.704
		Chemical	0.004	-0.111	0.118	0.062	0.951
		Visual	0.008	-0.107	0.123	0.131	0.896
		Chemical & visual	-0.024	-0.138	0.091	-0.396	0.693
		Noon	-0.054	-0.109	0.001	-1.898	0.061
	Interaction	Frame – chemical	0.007	-0.057	0.071	0.222	0.825
		Frame – visual	-0.008	-0.072	0.056	-0.234	0.815
		Frame – chemical & visual	-0.060	-0.124	0.005	-1.801	0.075
**Femoralis treatment only individuals within 80 mm from the center**							
		**Parameter**	**Estimate**	**Lower CI**	**Upper CI**	***t*-value**	***p*-value**
1500 frames	Main effect	**Intercept**	**4.245**	**4.193**	**4.297**	**156.272**	**< 2*10^{-16}**
		Frame	0.006	-0.015	0.027	0.571	0.570
		Chemical	-0.020	-0.012	0.075	-0.410	0.683
		Visual	-0.022	-0.102	0.059	-0.514	0.609
		Chemical & visual	0.031	-0.056	0.118	0.678	0.500
		Noon	-0.035	-0.092	0.021	-1.201	0.234
	Interaction	Frame – chemical	0.025	-0.020	0.070	1.082	0.283
		Frame – visual	0.015	-0.023	0.053	0.771	0.444
		Frame – chemical & visual	0.018	-0.024	0.060	0.833	0.408
3000 frames	Main effect	**Intercept**	**4.238**	**4.171**	**4.305**	**121.750**	**< 2*10^{-16}**
		Frame	-0.022	-0.062	0.018	-1.055	0.295
		Chemical	0.017	-0.011	0.144	0.250	0.803
		Visual	0.005	-0.103	0.113	0.090	0.929

		Chemical & visual	0.069	-0.048	0.186	1.133	0.261
		Noon	-0.050	-0.113	0.013	-1.537	0.130
	Interaction	Frame – chemical	0.061	-0.025	0.147	1.375	0.174
		Frame – visual	0.041	-0.031	0.114	1.094	0.278
		Frame – chemical & visual	0.051	-0.028	0.130	1.244	0.218
4500 frames	Main effect	**Intercept**	**4.264**	**4.188**	**4.340**	**107.075**	**< 2*10^{-16}**
		Frame	0.012	-0.026	0.051	0.619	0.538
		Chemical	0.002	-0.145	0.150	0.030	0.976
		Visual	-0.023	-0.148	0.102	-0.357	0.723
		Chemical & visual	0.033	-0.103	0.169	0.472	0.639
		Noon	-0.067	-0.135	-0.001	-1.918	0.060
	Interaction	Frame – chemical	0.013	-0.069	0.095	0.300	0.765
		Frame – visual	-0.014	-0.083	0.056	-0.380	0.705
		Frame – chemical & visual	-0.027	-0.103	0.049	-0.688	0.494
**Dragonfly treatment all individuals**							
		**Parameter**	**Estimate**	**Lower CI**	**Upper CI**	***t*-value**	***p*-value**
1500 frames	Main effect	**Intercept**	**4.102**	**4.053**	**4.149**	**165.351**	**< 2*10^{-16}**
		Frame	-0.004	-0.025	0.017	-0.362	0.719
		Chemical	-0.009	-0.090	0.073	-0.202	0.840
		Visual	0.040	-0.042	0.121	0.941	0.350
		Chemical & visual	0.069	-0.013	0.150	1.638	0.105
		Noon	-0.023	-0.076	0.031	-0.826	0.411
	Interaction	Frame – chemical	-0.015	-0.058	0.028	-0.686	0.495
		Frame – visual	0.035	-0.008	0.077	1.576	0.119
		**Frame – chemical & visual**	**0.051**	**0.009**	**0.094**	**2.339**	**0.022**
3000 frames	Main effect	**Intercept**	**4.095**	**4.034**	**4.157**	**128.087**	**< 2*10^{-16}**
		Frame	-0.030	-0.066	0.006	-1.609	0.111
		Chemical	0.053	-0.054	0.160	0.960	0.340

		Visual	0.079	-0.028	0.186	1.433	0.155
		**Chemical & visual**	**0.121**	**0.015**	**0.228**	**2.194**	**0.031**
		Noon	-0.053	-0.118	0.012	-1.580	0.118
	Interaction	Frame – chemical	0.059	-0.013	0.130	1.584	0.117
		Frame – visual	0.034	-0.037	0.106	0.918	0.361
		Frame – chemical & visual	0.050	-0.021	0.122	1.360	0.177
4500 frames	Main effect	**Intercept**	**4.112**	**4.044**	**4.179**	**117.464**	**< 2*10^{-16}**
		Frame	0.006	-0.024	0.035	0.368	0.714
		Chemical	0.044	-0.072	0.160	0.738	0.462
		Visual	0.079	-0.037	0.194	1.307	0.195
		**Chemical & visual**	**0.135**	**0.019**	**0.250**	**2.242**	**0.028**
		Noon	-0.060	-0.132	0.013	-1.591	0.115
	Interaction	Frame – chemical	0.014	-0.045	0.074	0.463	0.645
		Frame – visual	0.012	-0.048	0.071	0.376	0.708
		Frame – chemical & visual	0.031	-0.029	0.090	0.996	0.322
**Dragonfly treatment only individuals within 80 mm from the center**							
		**Parameter**	**Estimate**	**Lower CI**	**Upper CI**	***t*-value**	***p*-value**
1500 frames	Main effect	**Intercept**	**4.123**	**4.061**	**4.185**	**127.381**	**< 2*10^{-16}**
		Frame	0.006	-0.016	0.029	0.549	0.585
		Chemical	-0.016	-0.127	0.096	-0.269	0.789
		Visual	0.046	-0.068	0.159	0.772	0.443
		Chemical & visual	0.091	-0.017	0.198	1.610	0.113
		Noon	-0.018	-0.087	0.050	-0.511	0.611
	Interaction	Frame – chemical	-0.022	-0.070	0.025	-0.899	0.373
		Frame – visual	0.047	-0.001	0.094	1.888	0.064
		**Frame – chemical & visual**	**0.056**	**0.010**	**0.102**	**2.364**	**0.022**
3000 fram	Main effec	**Intercept**	**4.112**	**4.034**	**4.190**	**100.938**	**< 2*10^{-16}**
		Frame	-0.026	-0.071	0.018	-1.143	0.258

Journal of Experimental Biology • Supplementary information

		Chemical	0.052	-0.091	0.195	0.701	0.486
		Visual	0.113	-0.032	0.257	1.497	0.140
		**Chemical & visual**	**0.142**	**0.004**	**0.280**	**1.976**	**0.053**
		Noon	-0.030	-0.113	0.053	-0.699	0.487
	Interaction	Frame – chemical	0.070	-0.025	0.164	1.425	0.160
		Frame – visual	0.083	-0.011	0.178	1.700	0.095
		Frame – chemical & visual	0.062	-0.029	0.152	1.311	0.195
4500 frames	Main effect	**Intercept**	**4.135**	**4.051**	**0.176**	**94.134**	**< 2*10^{-16}**
		Frame	0.012	-0.024	0.047	0.622	0.536
		Chemical	0.055	-0.098	0.208	0.688	0.494
		Visual	0.128	-0.027	0.283	1.585	0.118
		**Chemical & visual**	**0.161**	**0.014**	**0.308**	**2.094**	**0.041**
		Noon	-0.041	-0.132	0.050	-0.854	0.400
	Interaction	Frame – chemical	0.033	-0.043	0.109	0.831	0.410
		Frame – visual	0.047	-0.029	0.124	1.193	0.238
		Frame – chemical & visual	0.042	-0.031	0.116	1.111	0.272
**Tinctorius treatment all individuals**							
		**Parameter**	**Estimate**	**Lower CI**	**Upper CI**	***t*-value**	***p*-value**
1500 frames	Main effect	**Intercept**	**4.198**	**4.1.54**	**4.241**	**185.181**	**< 2*10^{-16}**
		Frame	-0.004	-0.032	0.025	-0.240	0.811
		Chemical	-0.005	-0.082	0.072	-0.133	0.894
		Visual	-0.010	-0.087	0.067	-0.260	0.795
		Chemical & visual	-0.022	-0.099	0.055	-0.562	0.576
		Noon	-0.022	-0.063	0.019	-1.030	0.306
	Interaction	Frame – chemical	-0.015	-0.071	0.042	-0.514	0.608
		Frame – visual	0.009	-0.047	0.066	0.324	0.747
		Frame – chemical & visual	0.020	-0.037	0.076	0.670	0.505
3000	Mai	**Intercept**	**4.190**	**4.118**	**4.261**	**113.762**	**< 2*10^{-16}**

		Frame	-0.026	-0.072	0.019	-1.125	0.264
		Chemical	-0.009	-0.141	0.124	-0.128	0.899
		Visual	0.018	-0.115	0.150	0.258	0.797
		Chemical & visual	-0.026	-0.159	0.107	-0.381	0.704
		Noon	-0.044	-0.095	0.008	-1.651	0.102
	Interaction	Frame – chemical	0.009	-0.082	0.100	0.186	0.853
		Frame – visual	0.039	-0.052	0.129	0.823	0.413
		Frame – chemical & visual	0.009	-0.082	0.100	0.186	0.853
4500 frames	Main effect	**Intercept**	**4.213**	**4.132**	**4.293**	**100.736**	**< $2*10^{-16}$**
		Frame	0.006	-0.029	0.042	0.339	0.735
		Chemical	-0.032	-0.180	0.117	-0.410	0.683
		Visual	-0.002	-0.150	0.147	-0.024	0.981
		Chemical & visual	-0.038	-0.187	0.110	-0.499	0.619
		**Noon**	**-0.065**	**-0.127**	**-0.002**	**-1.987**	**0.050**
	Interaction	Frame – chemical	-0.024	-0.095	0.047	-0.660	0.511
		Frame – visual	-0.007	-0.078	0.064	-0.193	0.848
		Frame – chemical & visual	-0.006	-0.077	0.065	-0.162	0.871
**Tinctorius treatment only individuals within 80 mm from the center**							
		**Parameter**	**Estimate**	**Lower CI**	**Upper CI**	***t*-value**	***p*-value**
1500 frames	Main effect	**Intercept**	**4.224**	**4.168**	**4.280**	**144.144**	**< $2*10^{-16}$**
		Frame	0.006	-0.030	0.043	0.331	0.742
		Chemical	-0.019	-0.120	0.083	-0.353	0.725
		Visual	-0.002	-0.097	0.093	-0.039	0.969
		Chemical & visual	-0.013	-0.118	0.094	-0.226	0.822
		Noon	-0.029	-0.083	0.024	-1.055	0.296
	Interaction	Frame – chemical	-0.035	-0.109	0.039	-0.908	0.368
		Frame – visual	0.010	-0.060	0.079	0.264	0.793
		Frame – chemical & visual	0.027	-0.051	0.104	0.663	0.510

Journal of Experimental Biology • Supplementary information

3000 frames	Main effect	**Intercept**	**4.215**	**4.122**	**4.307**	**87.740**	**< $2*10^{-16}$**
		Frame	-0.023	-0.082	0.037	-0.735	0.465
		Chemical	-0.035	-0.213	0.142	-0.383	0.703
		Visual	0.036	-0.130	0.201	0.413	0.681
		Chemical & visual	0.024	-0.161	0.209	0.247	0.806
		Noon	-0.040	-0.105	0.025	-1.181	0.242
	Interaction	Frame – chemical	-0.005	-0.126	0.116	-0.079	0.938
		Frame – visual	0.048	-0.065	0.161	0.824	0.413
		Frame – chemical & visual	0.047	-0.079	0.173	0.724	0.472
4500 frames	Main effect	**Intercept**	**4.239**	**4.132**	**4.245**	**76.370**	**< $2*10^{-16}$**
		Frame	0.012	-0.036	0.060	0.492	0.624
		Chemical	-0.056	-0.260	0.148	-0.527	0.600
		Visual	0.014	-0.177	0.204	0.137	0.891
		Chemical & visual	-0.011	-0.223	0.202	-0.098	0.922
		Noon	-0.054	-0.131	0.023	-1.347	0.183
	Interaction	Frame – chemical	-0.030	-0.128	0.068	-0.584	0.562
		Frame – visual	-0.008	-0.100	0.084	-0.169	0.866
		Frame – chemical & visual	-0.011	-0.113	0.091	-0.211	0.834
**CUMULATIVE DISTANCE TRAVELLED**							
**Femoralis treatment**							
		**Parameter**	**Estimate**	**Lower CI**	**Upper CI**	***t*-value**	***p*-value**
1500 frames	Main effect	**Intercept**	**12.375**	**10.410**	**14.341**	**12.122**	**< $2*10^{-16}$**
		**Frame**	**4.330**	**3.534**	**5.125**	**10.482**	**< $2*10^{-16}$**
		Chemical	3.260	0.087	6.432	1.978	0.051
		**Visual**	**6.437**	**3.264**	**9.610**	**3.906**	**0.0002**
		Chemical & visual	-1.682	-4.854	1.491	-1.020	0.310
		Noon	-0.681	-3.019	1.658	-0.560	0.577
	Interact	Frame – chemical	0.725	-0.559	2.008	1.087	0.280

Journal of Experimental Biology • Supplementary information

		**Frame – visual**	**1.678**	**0.395**	**2.962**	**2.517**	**0.037**
		Frame – chemical & visual	-0.468	-1.752	0.816	-0.702	0.485
		Frame – noon	0.361	-0.585	1.307	0.735	0.464
3000 frames	Main effect	**Intercept**	**18.260**	**15.804**	**20.715**	**14.317**	**< $2*10^{-16}$**
		**Frame**	**6.881**	**5.890**	**7.873**	**13.365**	**< $2*10^{-16}$**
		Chemical	3.262	-0.702	7.225	1.584	0.117
		**Visual**	**7.112**	**3.149**	**11.076**	**3.455**	**0.001**
		Chemical & visual	-2.117	-6.081	1.846	-1.028	0.307
		Noon	-0.046	-2.966	2.875	-0.030	0.976
	Interaction	Frame – chemical	0.134	-1.466	1.734	0.161	0.872
		Frame – visual	1.024	-0.576	2.625	1.232	0.221
		Frame – chemical & visual	-0.504	-2.104	1.097	-0.606	0.546
		Frame – noon	0.675	-0.504	1.855	1.103	0.273
4500 frames	Main effect	**Intercept**	**22.952**	**20.138**	**25.765**	**15.706**	**< $2*10^{-16}$**
		**Frame**	**8.717**	**7.560**	**9.873**	**14.510**	**< $2*10^{-16}$**
		Chemical	3.140	-1.401	7.682	1.331	0.187
		**Visual**	**7.775**	**3.233**	**12.317**	**3.296**	**0.001**
		Chemical & visual	-2.059	-6.600	2.483	-0.873	0.385
		Noon	0.140	-3.207	3.487	0.080	0.936
	Interaction	Frame – chemical	-0.123	-1.990	1.744	-0.127	0.899
		Frame – visual	1.233	-0.634	3.101	1.272	0.207
		Frame – chemical & visual	-0.114	-1.981	1.753	-0.117	0.907
		Frame – noon	0.506	-0.870	1.882	0.708	0.481
**Dragonfly treatment**							
		**Parameter**	**Estimate**	**Lower CI**	**Upper CI**	***t*-value**	***p*-value**
1500 fram	Main effec	**Intercept**	**11.730**	**9.349**	**14.111**	**9.485**	**$5.64*10^{-15}$**
		**Frame**	**4.209**	**3.311**	**5.107**	**9.023**	**$4.85*10^{-14}$**

Journal of Experimental Biology • Supplementary information

		Chemical	2.929	-0.862	6.719	1.487	0.141
		Visual	2.043	-1.747	5.834	1.038	0.302
		Chemical & visual	1.969	-1.822	5.760	1.000	0.320
		Noon	0.581	-2.427	3.589	0.372	0.711
	Interaction	Frame – chemical	0.512	-0.918	1.941	0.689	0.493
		Frame – visual	0.454	-0.976	1.883	0.611	0.543
		Frame – chemical & visual	0.378	-1.051	1.808	0.509	0.612
		Frame – noon	0.597	-0.537	1.732	1.013	0.314
3000 frames	Main effect	**Intercept**	**6.384**	**5.610**	**7.157**	**15.894**	**$< 2*10^{-16}$**
		**Frame**	**1.753**	**1.492**	**2.013**	**12.945**	**$< 2*10^{-16}$**
		Chemical	0.711	-0.520	1.942	1.112	0.269
		Visual	0.698	-0.533	1.929	1.092	0.278
		Chemical & visual	0.530	-0.701	1.761	0.829	0.409
		Noon	0.371	-0.606	1.348	0.732	0.466
	Interaction	Frame – chemical	-0.234	-0.649	0.181	-1.089	0.281
		Frame – visual	-0.064	-0.478	0.352	-0.295	0.769
		Frame – chemical & visual	-0.101	-0.516	0.314	-0.469	0.641
		**Frame – noon**	**0.406**	**0.077**	**0.736**	**2.376**	**0.020**
4500 frames	Main effect	**Intercept**	**7.428**	**6.621**	**8.234**	**17.734**	**$< 2*10^{-16}$**
		**Frame**	**2.034**	**1.775**	**2.293**	**15.118**	**$< 2*10^{-16}$**
		Chemical	0.572	-0.712	1.855	0.857	0.394
		Visual	0.678	-0.606	1.962	1.017	0.312
		Chemical & visual	0.503	-0.781	1.786	0.754	0.453
		Noon	0.603	-0.416	1.622	1.140	0.258
	Interaction	Frame – chemical	-0.264	-0.676	0.149	-1.231	0.222
		Frame – visual	-0.053	-0.466	0.359	-0.248	0.804
		Frame – chemical & visual	-0.074	-0.487	0.338	-0.346	0.730

Journal of Experimental Biology • Supplementary information

		**Frame – noon**	**0.451**	**0.124**	**0.778**	**2.652**	**0.010**
		**Tinctorius treatment**					
		**Parameter**	**Estimate**	**Lower CI**	**Upper CI**	***t*-value**	***p*-value**
1500 frames	Main effect	**Intercept**	**11.710**	**9.484**	**13.937**	**10.126**	**< $2.87*10^{-16}$**
		**Frame**	**4.100**	**3.282**	**4.919**	**9.649**	**< $2.62*10^{-15}$**
		Chemical	2.453	-1.155	6.060	1.309	0.194
		Visual	0.449	-3.159	4.056	0.239	0.811
		Chemical & visual	0.135	-3.473	3.742	0.072	0.943
		Noon	0.621	-1.948	3.190	0.465	0.643
	Interaction	Frame – chemical	0.193	-1.133	1.519	0.280	0.780
		Frame – visual	0.092	-1.234	1.417	0.133	0.894
		Frame – chemical & visual	-0.272	-1.598	1.054	-0.395	0.694
		Frame – noon	0.809	-0.135	1.753	1.651	0.102
3000 frames	Main effect	**Intercept**	**17.191**	**14.483**	**19.898**	**12.223**	**< $2*10^{-16}$**
		**Frame**	**6.431**	**5.419**	**7.443**	**12.236**	**< $2*10^{-16}$**
		Chemical	2.202	-2.185	6.590	0.966	0.337
		Visual	-0.071	-4.458	4.316	-0.031	0.975
		Chemical & visual	0.164	-4.223	4.552	0.072	0.943
		Noon	2.046	-1.078	5.170	1.261	0.211
	Interaction	Frame – chemical	-0.202	-1.842	1.437	-0.237	0.813
		Frame – visual	-0.543	-2.182	1.097	-0.637	0.526
		Frame – chemical & visual	-0.031	-1.670	1.609	-0.036	0.971
		**Frame – noon**	**1.557**	**0.389**	**2.723**	**2.567**	**0.012**
4500 frames	Main effect	**Intercept**	**21.650**	**18.579**	**24.722**	**13.570**	**< $2*10^{-16}$**
		**Frame**	**8.247**	**7.087**	**9.408**	**13.679**	**< $2*10^{-16}$**
		Chemical	2.110	-2.867	7.087	0.816	0.417
		Visual	-0.540	-5.517	4.437	-0.209	0.835
		Chemical & visual	0.453	-4.525	5.430	0.175	0.861

Journal of Experimental Biology • Supplementary information

		Noon	2.686	-0.859	6.230	1.459	0.148
	Interaction	Frame – chemical	-0.211	-2.092	1.670	-0.216	0.830
		Frame – visual	-0.810	-2.691	1.072	-0.829	0.410
		Frame – chemical & visual	0.342	-1.539	2.223	0.350	0.727
		**Frame – noon**	**1.424**	**0.085**	**2.764**	**2.047**	**0.044**

Table S2. Results from the Kruskal-Wallis test looking at consistency in the cumulative distance moved towards/ away from the centre and the cumulative distance travelled after 1500, 3000 and 4500 frames in the femoralis, dragonfly and tinctorius treatment.

**Cumulative distance moved towards/ away from the centre**				
		**Chi²**	**df**	***p*-value**
Femoralis	1500	0.780	3	0.854
	3000	0.729	3	0.866
	4500	3.466	3	0.325
Dragonfly	1500	4.645	3	0.200
	3000	2.192	3	0.534
	4500	2.031	3	0.566
Tinctorius	1500	1.529	3	0.676
	3000	0.922	3	0.820
	4500	0.992	3	0.803
**Cumulative distance travelled**				
		**Chi²**	**df**	***p*-value**
Femoralis	**1500**	**13**	**3**	**0.005**
	**3000**	**9.018**	**3**	**0.029**
	4500	6.781	3	0.079
Dragonfly	1500	1.696	3	0.638

Journal of Experimental Biology • Supplementary information

	3000	1.296	3	0.730
	4500	0.985	3	0.805
Tinctorius	1500	0.572	3	0.903
	3000	0.151	3	0.985
	4500	0.846	3	0.839
